# Erlotinib-induced complete response in a patient with *epidermal growth factor receptor* wild-type lung adenocarcinoma after chemotherapy failure: a case report

**DOI:** 10.1186/1752-1947-8-102

**Published:** 2014-03-24

**Authors:** Maria Giuseppa Vitale, Ferdinando Riccardi, Carmela Mocerino, Carmela Barbato, Roberto Monaco, Paola Galloro, Nicola Gagliardi, Giacomo Cartenì

**Affiliations:** 1 UOSC Oncologia, Azienda Ospedaliera di Rilievo Nazionale “Antonio Cardarelli”, Naples, Italy; 2 UOSC Anatomia Patologica, Azienda Ospedaliera di Rilievo Nazionale “Antonio Cardarelli”, Naples, Italy; 3 UOSC Radiologia, Azienda Ospedaliera di Rilievo Nazionale “Antonio Cardarelli”, Naples, Italy

**Keywords:** *EGFR* amplification, *EGFR* mutation, erlotinib, Second-line treatment in NSCLC

## Abstract

**Introduction:**

The efficacy of erlotinib in advanced non-small-cell lung cancer has been demonstrated in several trials, but only two cases of complete and prolonged response in wild-type epidermal growth factor receptor locally advanced lung cancer have been published.

**Case presentation:**

We discuss a case of a 67-year-old Caucasian man, a former heavy cigarette smoker, with a diagnosis of wild-type epidermal growth factor receptor locally advanced adenocarcinoma. After platinum-based doublet chemotherapy, when a progression of disease had occurred, a second-line therapy with erlotinib was started. We observed a progressive reduction of his lung lesion during erlotinib treatment until there was a complete clinical response.

**Conclusions:**

This case is interesting for the choice of second-line treatment in non-small-cell lung cancer and, moreover, for the possibility of a complete and prolonged response to erlotinib even in patients without the activating mutation of epidermal growth factor receptor.

## Introduction

Lung cancer is one of the most common types of cancer and the leading cause of human cancer deaths worldwide [1]. The majority of the cases (85%) are classified as non-small-cell lung cancer (NSCLC), often diagnosed at an advanced stage with poor prognosis. Platinum-based doublet chemotherapy has become the standard of care for the treatment of advanced or metastatic NSCLC with wild-type or unknown epidermal growth factor receptor (EGFR) status [2].

EGFR-targeted therapy constitutes a new treatment opportunity.

The EGFR tyrosine kinase inhibitors (TKIs) gefitinib and erlotinib block signal transduction pathways and inhibit cancer cell survival and proliferation. NSCLC with activating mutations of the EGFR tyrosine kinase are highly sensitized to the effects of oral TKIs.

Erlotinib has demonstrated efficacy in the first-line treatment of *EGFR* mutation-positive NSCLC [3,4]. Several clinical trials have also demonstrated that erlotinib improves progression-free survival (PFS) and overall survival (OS) in the second- and third-line treatment of NSCLC [5] as well as in the maintenance setting [6]. It is known that a mutation in the *EGFR* gene, clinical characteristics like female sex, non-smoking status and Asian ethnicity, adenocarcinoma histology and skin toxicity reported during the treatment, give an increased response to EGFR inhibitors.

Here, we describe a complete response to erlotinib treatment in a male former heavy cigarette smoker with wild-type *EGFR* adenocarcinoma. Therefore, it appears that treatment with the TKI erlotinib may possibly confer a benefit in terms of PFS and OS, regardless of *EGFR* mutation status and other clinical features.

## Case presentation

**Figure 1:**
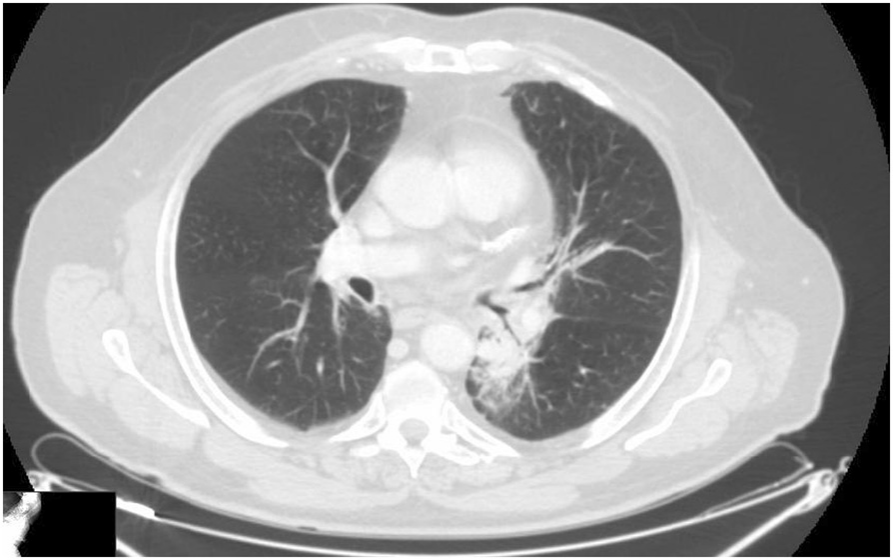
Computed tomography scan performed before starting erlotinib treatment.

**Figure 2:**
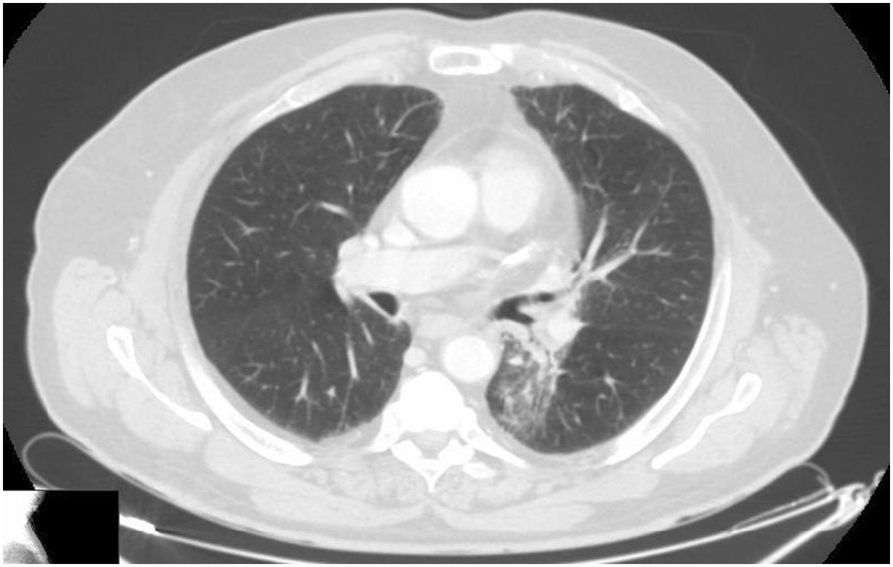
First evaluation performed after 4 months of treatment with erlotinib.

**Figure 3:**
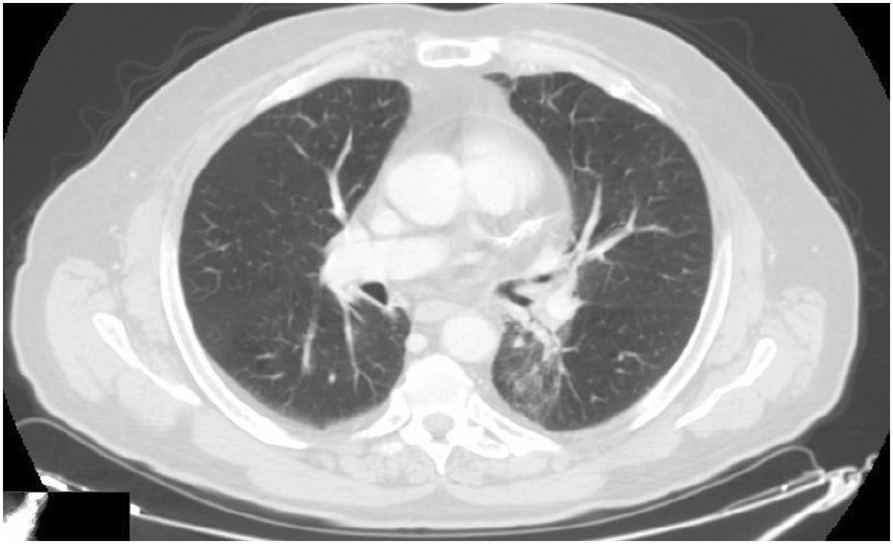
A computed tomography scan performed 8 months after starting treatment with erlotinib.

**Figure 4:**
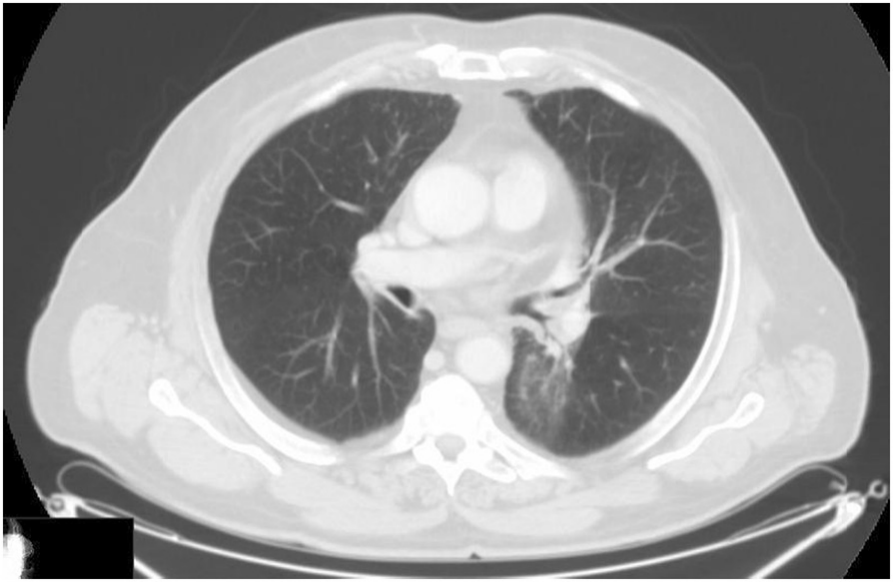
A computed tomography scan performed 1 year after starting treatment with erlotinib and showing a complete response.

A 67-year-old Caucasian man, a former heavy cigarette smoker, presented to our hospital with a persistent cough. He had a medical history of ischemic heart disease, chronic obstructive pulmonary disease (COPD) and arterial hypertension. A whole-body computed tomography (CT) scan demonstrated a solid nodule with speculated margins, measuring approximately 33×33mm, in the apical segment of his left lower lobe and an infiltration of the pleura and of pulmonary vessels. Enlarged ipsilateral peribronchial lymph nodes (24mm) and some small lymph nodes of the aortopulmonary window were also described. A bronchoscope image showed only hyperemic inflamed mucosa and cytology did not provide a cancer diagnosis. Therefore, a CT-guided fine-needle aspiration biopsy of his pulmonary nodule was performed. Histology and mutation testing revealed that the tumor was a wild-type *EGFR* adenocarcinoma (thyroid transcription factor-1 positive). A radical surgery of the lung lesion was excluded because of a chronic obstructive pulmonary disease. The patient started a first-line cisplatin-gemcitabine chemotherapy obtaining a partial response after six courses: a whole body CT scan revealed a reduction of his lung lesion (12mm versus 33mm) and of peribronchial lymph nodes (18mm versus 24mm). In view of the inoperability, he was subsequently treated with radiation therapy gaining a further reduction of the lung lesion and metastatic lymph nodes. However, 8 months later, a CT scan established disease progression: the lung nodule increased to 19 mm (Figure 1), whereas his lymph nodes appeared stable. The short disease progression-free interval and the clinical history of this patient ruled out the use of a new chemotherapy; a second-line therapy with erlotinib at a dose of 150mg/day was started May 2012. A CT scan performed after 4 months of erlotinib treatment showed a reduction of pulmonary lesion volume (Figure 2) and the treatment was continued. Four months later, a new CT scan exhibited a significant reduction of the tumor in the lower lobe of his left lung (7mm; Figure 3). Finally, in the last CT scan an area of parenchymal dysventilation in the apical segment of the lower lobe of his left lung was recognized, while the neoplastic mass was no longer visible (Figure 4), confirming a complete remission after 1 year of the erlotinib therapy. In view of the surprising response to TKI treatment, we also performed a fluorescence *in situ* hybridization (FISH) analysis for *EGFR*, but this test did not reveal a gene amplification or high polysomy. The treatment with erlotinib was well tolerated; in fact, the only adverse event was a grade 1 skin rash on his face, which occurred early in the treatment and regressed after 4 weeks with a topical therapy. At the time that this article was revised, our patient was still undergoing treatment and he was free of disease.

## Discussion

Lung cancer is often diagnosed after development of the late stage disease [7] and palliative chemotherapy is associated with a modest survival benefit and improved quality of life (QoL) [8,9]. Platinum-based doublet chemotherapy (with agents such as gemcitabine, taxanes, vinorelbine, pemetrexed and bevacizumab) represents the standard of care for the treatment of locally advanced or metastatic NSCLC with wild-type or unknown epidermal growth factor receptor (EGFR) status [2]. During recent years, the study of the molecular characteristics of NSCLC has highlighted the specific role of certain genes. The Erb family of cell membrane receptors comprises the EGFR which is involved in cell proliferation, differentiation, and survival [10]. Activating *EGFR* mutations (exon 19 deletion or exon 21 substitution) are predictive for response to the EGFR-TKIs, gefitinib and erlotinib. These molecules, ultimately approved for the treatment of advanced NSCLC, have changed the clinical outcome of these malignancies. The incidence of *EGFR* mutations in the Caucasian population is approximately 10% and higher still in never-smokers, in the adenocarcinoma subtype, and in women. In comparison, the prevalence of this mutation is the highest in East Asian patients (approximately 40%). Several randomized trials have shown that TKIs provide an improved response rate (RR) and PFS, exhibiting a good tolerability of treatment and a better QoL, when compared with chemotherapy as first-line therapy. Indeed, EGFR-TKIs represent the standard first-line treatment for *EGFR*-mutated advanced NSCLC [3,4].

Although *EGFR* mutations can account for most of the responses to EGFR-TKIs therapy, the clinical benefits cannot only be explained by these mutations. Therefore, closer care should be delivered to patients with wild-type *EGFR* with the aim to determine whether or when they should receive EGFR-TKI treatment. The *EGFR* gene, in fact, may undergo polysomy and/or amplification, as detected by FISH. In addition to mutated *EGFR*, there is now evidence that increased *EGFR* gene copy number, as defined as amplification (ratio≥2, ≥15 copies of *EGFR* gene in ≥10% of the cells, the *EGFR* gene clusters) or high *EGFR* polysomy (≥4 copies of the *EGFR* gene in ≥40% of cells), is associated with a better response to gefitinib and erlotinib [11,12].

The results from the BR.21 trial [5,13] suggested that patients with increased *EGFR* gene copy number may have longer survival (BR. 21: hazard ratio, HR, 0.43, 95% confidence interval 0.23 to 0.78, p<0.004). Therefore, further research is needed to refine the potential impact of the number of mutated copies of the *EGFR* gene as the factor modifying prospective benefits of TKI treatment.

Regarding second-line therapy, treatment options consist of pemetrexed [14] limited to the non-squamous histology, docetaxel [15] or erlotinib [5]. The BR.21 trial was designed to compare erlotinib (in second- and third-line treatment or patients not eligible for further chemotherapy) with best supportive care in 731 patients with advanced NSCLC. In this study, patients treated with erlotinib had a RR of 8.9%, a statistically significant increase in median (6.7 versus 4.7 months) and 1-year survival rate (31% versus 21%) compared to the patients receiving a placebo, and this benefit was similar across multiple subgroups. Moreover, smoking status appeared to be the most powerful predictor of a survival effect with erlotinib: never-smokers receiving erlotinib had a significantly higher survival rate than patients in the placebo arm (HR, 0.4; p <0.01) [16]. This randomized trial demonstrated erlotinib can prolong survival after chemotherapy and can give significant benefits in QoL and lung cancer-related symptoms of cough, dyspnea and pain [13]. The safety and efficacy profile of erlotinib has been confirmed by a phase IV study (Tarceva Lung Cancer Survival Treatment (TRUST)) in a heterogeneous NSCLC population including more than 6500 patients. This trial included patients with advanced stage IIIB/IV NSCLC who had previously failed on or were considered unsuitable to receive standard chemotherapy or radiotherapy and were ineligible for other erlotinib trials. In patients with advanced NSCLC, the PFS and OS in this study were 3.25 months and 7.9 months, respectively, and the disease control rate was 69%. Results from the TRUST study suggested that erlotinib can benefit a wide range of patients, including those who have previously been thought unlikely to respond to this treatment [17].

Previous data have been reinforced, finally, by the TITAN trial showing that erlotinib is equivalent to pemetrexed or docetaxel in refractory patients progressing during the four cycles of a standard platinum-based chemotherapy doublet [18]. However, more recently, the TAILOR trial results show that chemotherapy is more effective than erlotinib for second-line treatment for previously treated patients with NSCLC who have wild-type *EGFR* tumors. Median OS (8.2 months with docetaxel versus 5.4 months with erlotinib) and PFS (2.9 versus 2.4) were significantly better with docetaxel than with erlotinib [19].

In this trial, only 3% of patients had a partial response to erlotinib, and it is possible that there are determinants other than *EGFR* mutations of erlotinib efficacy that have yet to be fully validated. Potential biomarkers include *EGFR* amplification and/or presence of EGFR ligands. However, given the low RR, these are likely to exist in only a small subset of *EGFR* wild-type NSCLC patients.

Although evidence of the TAILOR trial indicated the superiority of using chemotherapy in a second-line to treat patients who were *EGFR* mutation-negative, further study is required to verify that for patients who are *EGFR* FISH positive it may be favorable to choose EGFR-TKIs or chemotherapy treatment in *EGFR* wild-type population considering improved QoL and tolerable toxicity. A combined analysis of *EGFR* FISH and mutation is an effective predictor of EGFR-TKI therapy. Specifically, *EGFR* gene copy number should be further detected in patients with wild-type *EGFR*, because a high *EGFR* copy number may predict a greater benefit from TKIs treatment.

Erlotinib has a good safety profile and main toxicities are skin rash and diarrhea. Skin rash has been confirmed as an independent predictive factor for progression (HR 0.50, p<0.00001) and survival (HR 0.30, p<0.0001) in TKI-treated NSCLC patients. Patients who developed grade 2 to 4 skin rash were more likely to respond to the treatment (42%) compared to those who did not (7%). Thus, skin rash development has been strongly correlated with EGFR-TKI efficacy [20]. Our patient developed grade 1 skin rash that regressed completely after a month with topical therapy.

Therefore, the characteristics of this patient (Caucasian ethnicity, male gender, cigarette smoking history and no severe skin rash) did not represent the well-known predictors of best response to EGFR-TKIs.

Nevertheless, our patient has achieved not only a complete response, but he has also exceeded the median OS and PFS recorded in the BR.21 trial.

## Conclusions

To the best of our knowledge, there are only two cases described in the literature of a complete response to second-line therapy with erlotinib in *EGFR* wild-type NSCLC [21,22].

The TKI erlotinib confers a benefit in terms of RR, PFS and OS, in patients with NSCLC, and this benefit can appear even regardless of *EGFR* mutation status, clinical characteristics and skin toxicity.

In patients with progressive NSCLC after failure of standard chemotherapy, a second-line treatment with erlotinib should be considered in view of the good safety profile, easy administration and possibility of a long treatment period with a good QoL.

The choice of erlotinib at disease progression, instead of second-line chemotherapy, could be preferable for patients with co-morbidities and for patients which have a low probability of a response to a cytotoxic treatment.

In the present case report, the choice of erlotinib in second line of treatment exhibited a surprising response, because the clinical characteristics of our patient did not correlate with those predictive of response or benefit. Despite the negative *EGFR* mutational test and only a mild and transient skin rash, the clinical efficacy of the TKI continued for several months keeping a complete response.

The clinical response has been complete and prolonged. In fact, in the BR.21 trial the OS and the PFS have been 6.7 and 2.2 months, respectively, in the erlotinib-treated subset, therefore, our patient has already exceeded those periods of time gaining a survival benefit. Finally, from this we suggest that no assumptions should be made on the *EGFR* test in the choice of second-line treatment for advanced NSCLC.

Despite our knowledge, further studies are needed on the EGFR pathway, with focus on unknown pathways that could be altered and activated from a platinum-based chemotherapy.

## Consent

Written informed consent was obtained from the patient for publication of this case report and any accompanying images. A copy of the written consent is available for review by the Editor-in-Chief of this journal.

## Abbreviations

COPD: Chronic obstructive pulmonary disease; CT: Computed tomography; EGFR: Epidermal growth factor receptor; FISH: Fluorescence *in situ* hybridization; HR: Hazard ratio; NSCLC: Non-small-cell lung cancer; OS: Overall survival; PFS: Progression-free survival; QoL: Quality of life; RR: Response rate; TKIs: Tyrosine kinase inhibitors; TRUST: Tarceva Lung Cancer Survival Treatment.

## Competing interests

All the authors declare: “I have no competing interests”.

## Authors’ contributions

RM and PG carried out the histological finding and molecular genetic studies. NG carried out the radiological imaging. MGV and FR drafted the manuscript. MGV, FR, CM, CB and GC discussed the case, have been involved in the acquisition, analysis and interpretation of data and gave a specific contribution in the manuscript drafting. All authors read and approved the final manuscript.
